# Supplementary material for: Persistent viral infections impact key biological traits in Drosophila melanogaster
Source: PLoS Biol. 2025 Oct 9;23(10):e3003437. doi: 10.1371/journal.pbio.3003437 (PMC12530575; doi:10.1371/journal.pbio.3003437)
Supplement: S1 Table — (DOCX) [file pbio.3003437.s006.docx]

**S1 Table.** Primers used in qPCR step of RT-qPCR for viral load quantification.

| **Primer name** | **Sequence** |
| --- | --- |
| Rp49_Forward | CGGATCGATATGCTAAGCTGT |
| Rp49_Reverse | GCGCTTGTTCGATCCGTA |
| DAV_Forward | GTTGGATCAGGCTAGTGTAGG |
| DAV_Reverse | TGCAACCGGACTCCAAGTTC |
| DCV_Forward | AGGCTGTGTTTGCGCGAAG |
| DCV_Reverse | AATGGCAAGCGCACACAATTA |
| Bloomfield_Forward | CCGCCGGCTACCTACTTTAG |
| Bloomfield_Reverse | ACCAGCAAGCGTCCGAAATA |
| Nora_Forward | GGTTTGCAGTTGATCGCAG |
| Nora_Reverse | TCAGCTCATCGTTCACGAA |
| 8/27_Forward | AGAGTTTGATCMTGGCTCAG |
| 1492_Reverse | CGGTTACCTTGTTACGACTT |
| 16S_qPCR_Forward | TCCTACGGGAGGCAGCAGT |
| 16S_qPCR_Reverse | GGACTACCAGGGTATCTAATCCTGTT |
| Rp49_gDNA_Forward | GAAGTTCCTGGTGCACAACG |
| Rp49_gDNA_Reverse | CTTGCGCTTCTTGGAGGAGA |
