# Supplementary material for: Persistent viral infections impact key biological traits in Drosophila melanogaster
Source: PLoS Biol. 2025 Oct 9;23(10):e3003437. doi: 10.1371/journal.pbio.3003437 (PMC12530575; doi:10.1371/journal.pbio.3003437)
Supplement: S2 Table — (DOCX) [file pbio.3003437.s007.docx]

**S2 Table.** Primers used for RT-PCR to assess the presence of viruses and *Wolbachia*.

| **Primer name** | **Sequence** |
| --- | --- |
| DAV_Forward | AGAGTGGCTGTGAGGCAGAT |
| DAV_Reverse | GCCATCTGACAACAGCTTGA |
| DCV_Forward | GTTGCCTTATCTGCTCTG |
| DCV_Reverse | CGCATAACCATGCTCTTCTG |
| Nora_Forward | ATGGCGCCAGTTAGTGCAGACCT |
| Nora_Reverse | CCTGTTGTTCCAGTTGGGTTCGA |
| Bloomfield_Forward_Seg4 | CTATGGTTATCGATTGCATGGTCC |
| Bloomfield_Reverse_Seg4 | GTAAACAAATCAAAACCATC |
| Bloomfield_Forward_Seg7 | ATTTTTGGACTCAGATTGG |
| Bloomfield_Reverse_Seg7 | GCCAAAATACTTGTTCCAG |
| Wsp_Forward | TGGTCCAATAAGTGATGAAGAAAC |
| Wsp_Reverse | AAAAATTAAACGCTACTCCA |
| WspB_Forward | TTTGCAAGTGAAACAGAAGG |
| WspB_Reverse | GCTTTGCTGGCAAAATGG |
