# Supplementary figures and images for: Persistent viral infections impact key biological traits in Drosophila melanogaster
Source: PLoS Biol. 2025 Oct 9;23(10):e3003437. doi: 10.1371/journal.pbio.3003437 (PMC12530575; doi:10.1371/journal.pbio.3003437)

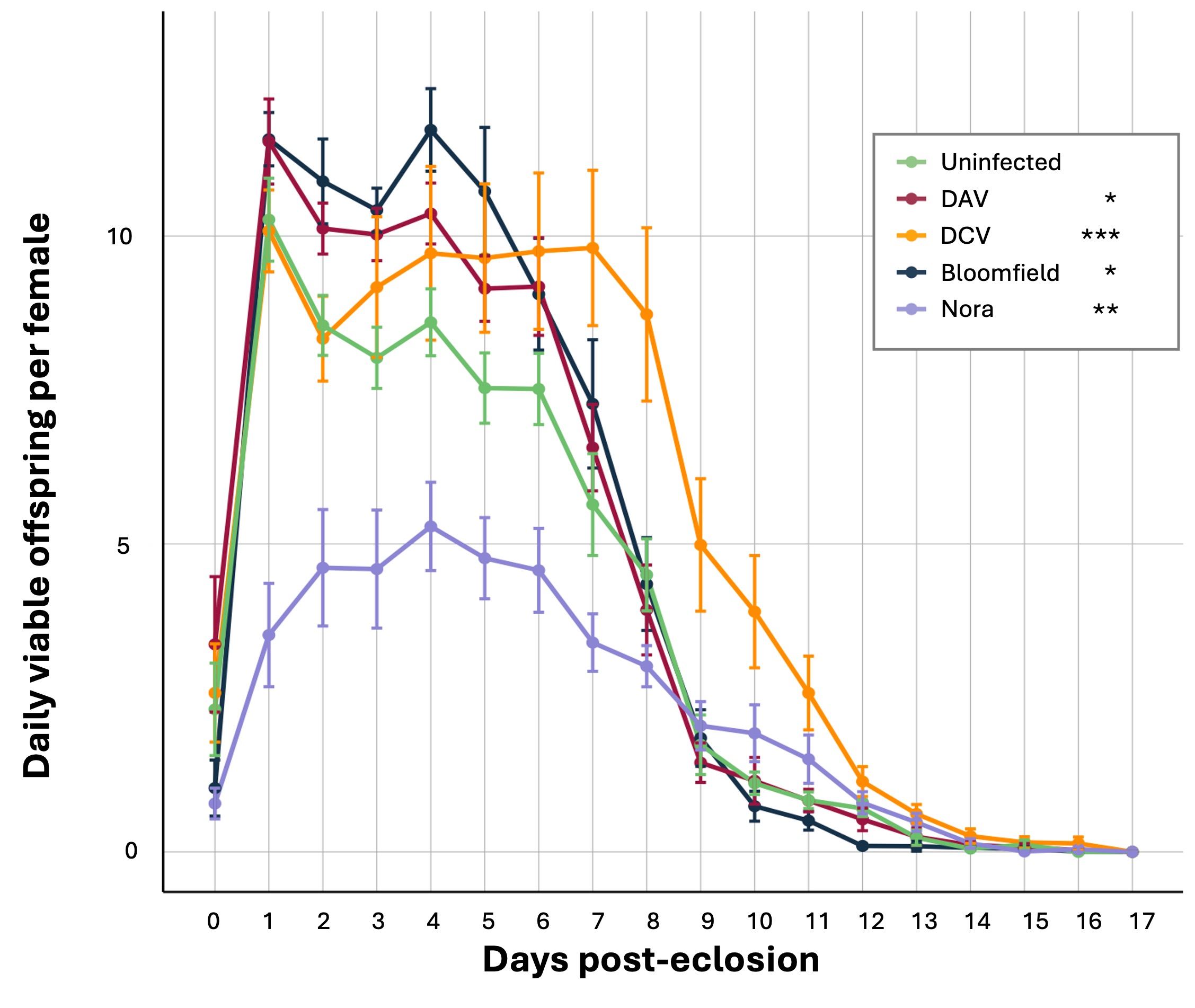

Supplement: S1 Fig — Daily viable offspring per female. Number of viable offspring produced by female flies uninfected (green) or persistently infected with DAV (red), DCV (yellow), Bloomfield virus (dark blue) or Nora virus (purple) after one day of mating (20 females paired with 20 males). After one day, the males were discarded, and the females were transferred to new vials with food each day. Mated flies were kept for subsequent days, and the viable offspring were counted. The number of viable offspring was normalized by the number of alive females eggs in each tube. Day 0 represents the day of eclosion and mating. Experiments were conducted on 20 females paired with 20 males in 9 replicates. Significance was calculated using a general linear-mixed model where the time point was the fixed factor and experiments a random effect; Tukey contrasts were used for post-hoc analyses. Significance values are *** P < 0.001, ** P < 0.01, * P < 0.05. (TIF) [file pbio.3003437.s008.tif]

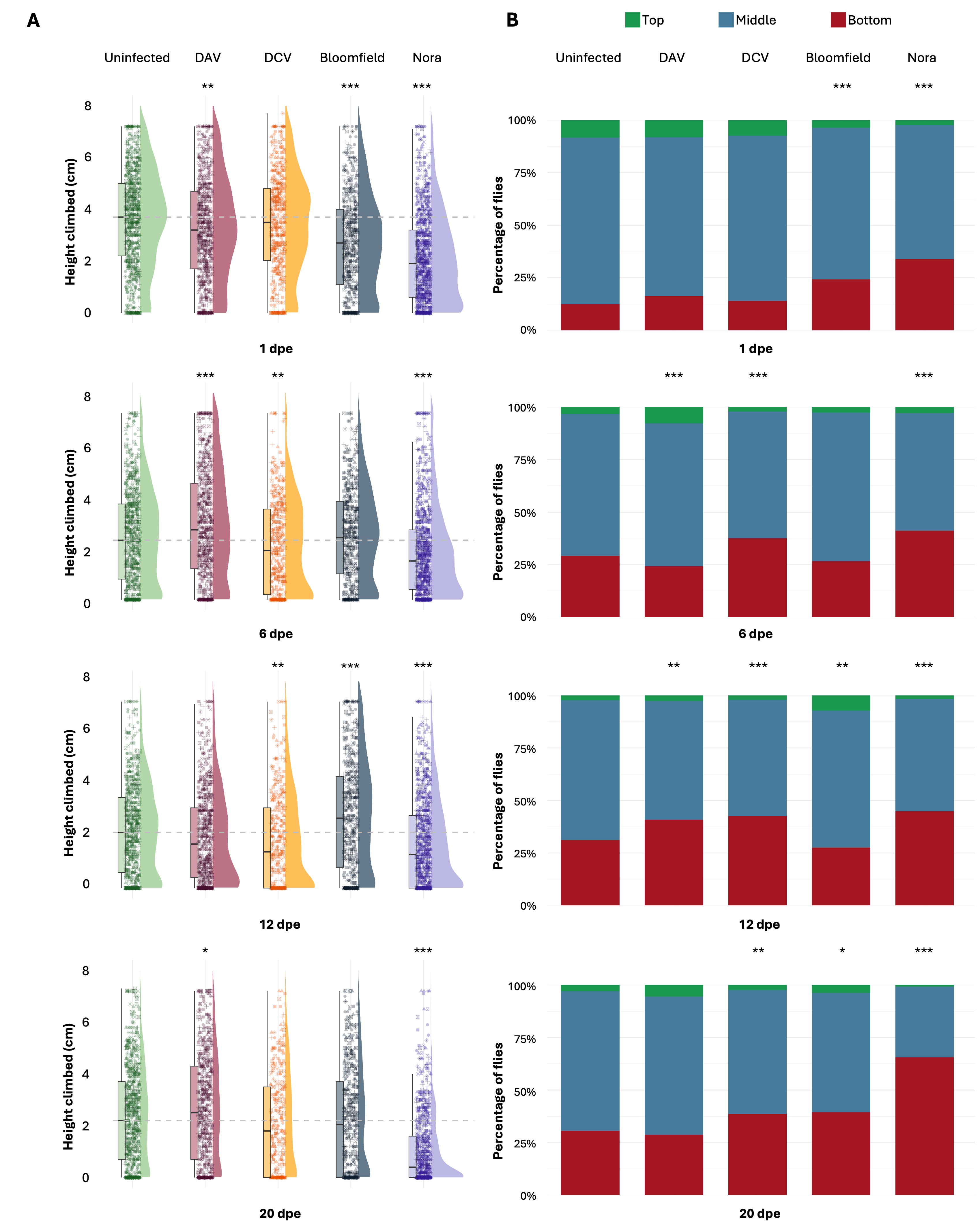

Supplement: S2 Fig — Climbing assay results of uninfected flies (green) or flies persistently infected with DAV (red), DCV (yellow), Bloomfield virus (dark blue) or Nora virus (purple). A) Quantitative height climbed by individual flies. Climbing assay results for days 1, 6, 12, and 20 post-eclosion. Results of 9 different biological replicates with 20 female flies. At each time point and replicate, six pictures were taken, and the height of each fly in the tube was recorded. Each symbol on the graph represents the recorded height of a fly for each picture. Boxplots are shown on the left side of each stock, indicating the median and interquartile range of the heights climbed by the flies. The dotted line represents the median height climbed by the uninfected population. The half-violin of each plot shows the density of the values. Significance was calculated using a general linear-mixed model where the virus status and the time point were the fixed factors and experiments a random effect; Tukey contrasts were used for post-hoc analyses. B) Qualitative measurements of the climbing assay. Top (green) indicates the flies present in the top 1 cm of the climbing tubes (6.2-7.2 cm). Bottom (red) indicates the percentage of flies located in the bottom 1 cm of the tube (0-1 cm), and middle (blue) includes all flies that climbed between (1-6.2 cm). Significance was calculated using a cumulative link mixed model where the virus status and the time point were the fixed factors and experiments a random effect; false discovery rate contrasts were used for post-hoc analyses. For all the figure, *** P < 0.001, ** P < 0.01, * P < 0.05. (TIF) [file pbio.3003437.s009.tif]

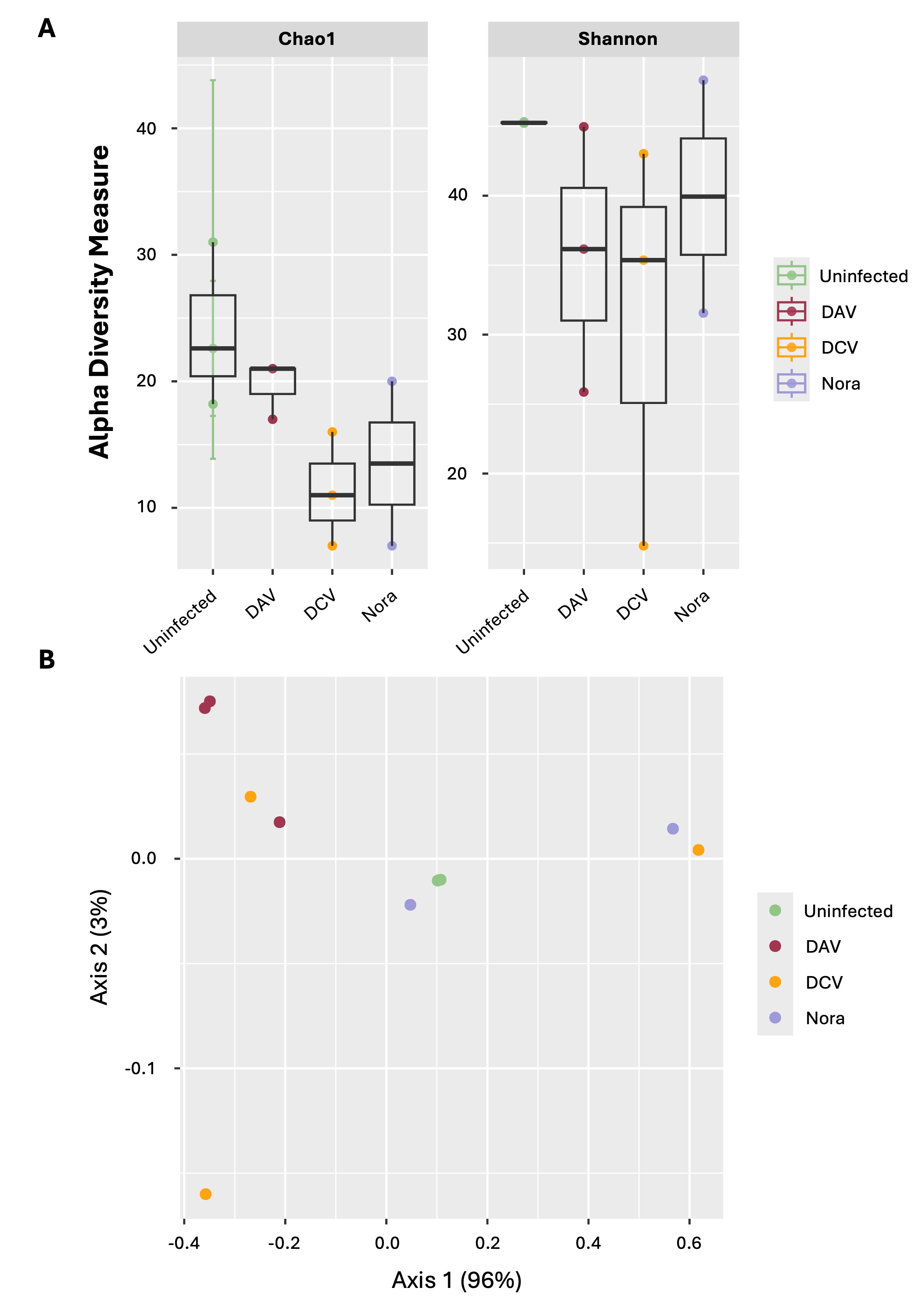

Supplement: S3 Fig — A) Alpha diversity analyses on Chao1 and Shannon indexes reveal no significant differences in the species diversity of uninfected or persistently infected flies (pairwise Wilcoxon test on Chao1 and Shannon indexes, P > 0.46 for all comparisons). B) Principal component analysis of the Bray-Curtis dissimilarity matrix of the different studied fly populations at the OTU level. This analysis does not identify any specific differences in the bacterial community structure among the various fly lines (PERMANOVA on infection, F = 1.57, P = 0.24). (TIF) [file pbio.3003437.s010.tif]

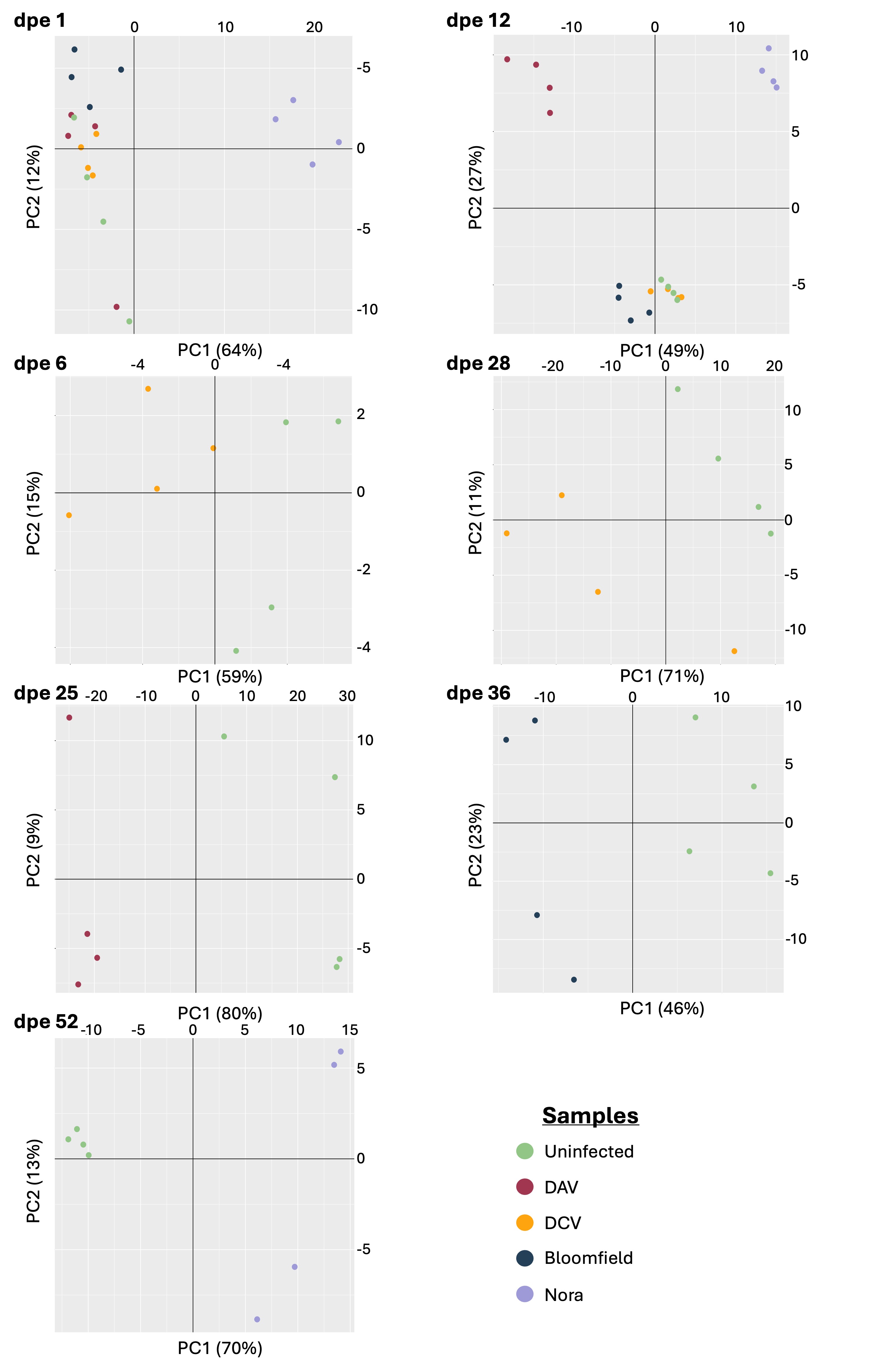

Supplement: S4 Fig — These samples consist on pools of RNA coming from individual Drosophila females from the uninfecetd and infected stocks. Infection was confirmed through RT-qPCR. All samples in a pool (3-5) come from the same collection dpe and stock. On the Y axis of every graph, we indicate the second principal component (PC2) and the percentage of variance explained by this component between brackets. On the X axis, we indicate the first principal component (PC1) and the percentage of variance explained by this component between brackets. Black lines indicate the origin of each axis. The do represent our fly stocks throughout the different days post-eclosion, in green, we have the Uninfected stock, in burgundy, the Drosophila A virus persistent flies, in yellow, the Drosophila C virus persistently infected flies, in dark blue, we have the Bloomfield virus persistently infected stock, and in lavender the Nora virus persistently infected flies. (TIF) [file pbio.3003437.s011.tif]

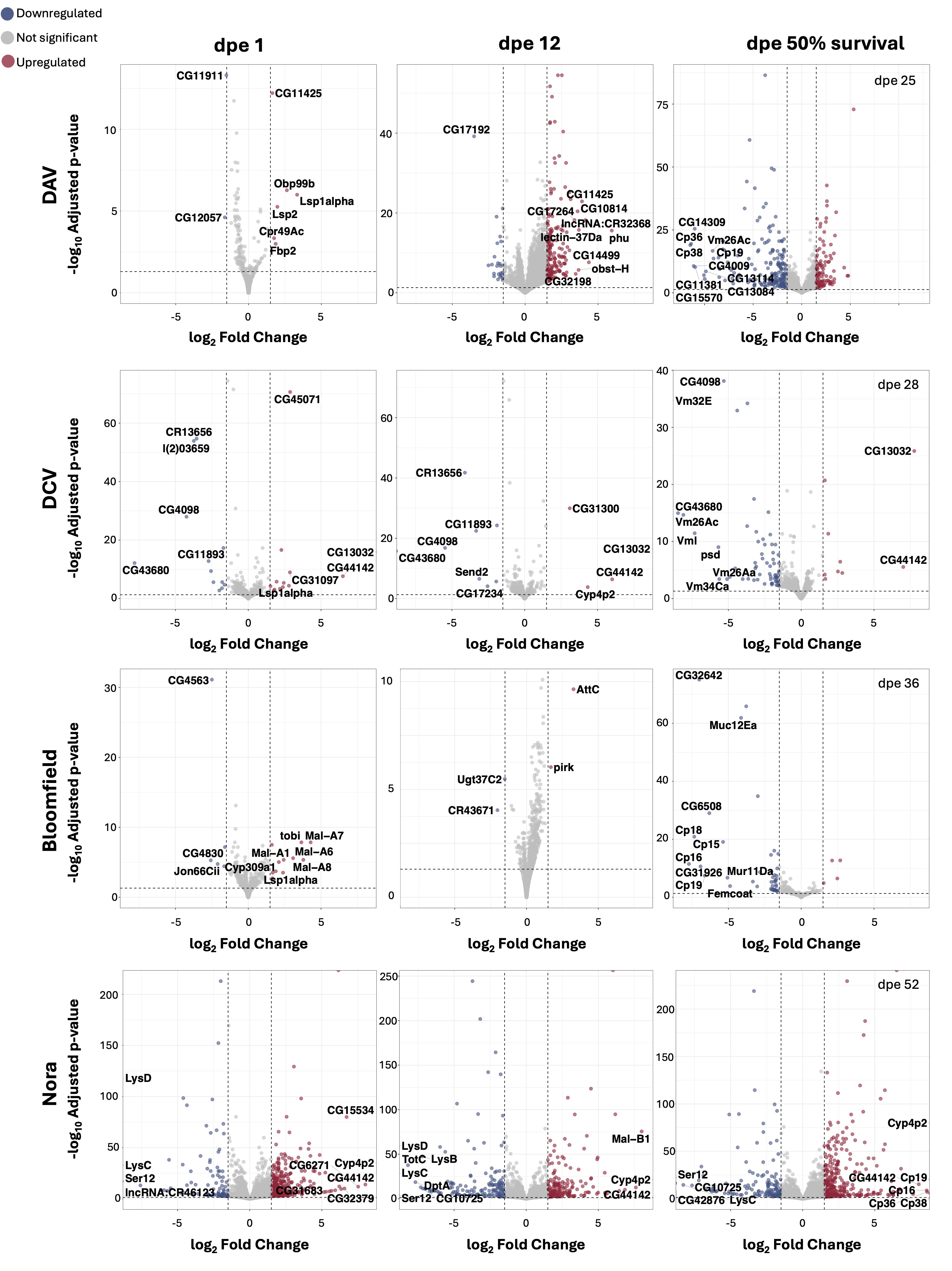

Supplement: S5 Fig — Transcriptomic results indicate differentially expressed genes in our infected samples. Volcano plots showing the number of genes significantly up- (in red) or down- (in blue) regulated. In grey, we represent those genes displaying differential expression values lower than 1.5 log2 fold change and/or with p-adjusted values lower than 0.05. Gene names are indicated fot the top 10 genes in absolute Log2FoldChange values. These thresholds are indicated by dotted lines in the graphs. Each row presents the volcano plots of a specific fly stock over time (dpe), in the following order: DAV, DCV, Bloomfield, and Nora. Each infected sample is analysed in comparison to an uninfected control of the same age. (TIF) [file pbio.3003437.s012.tif]

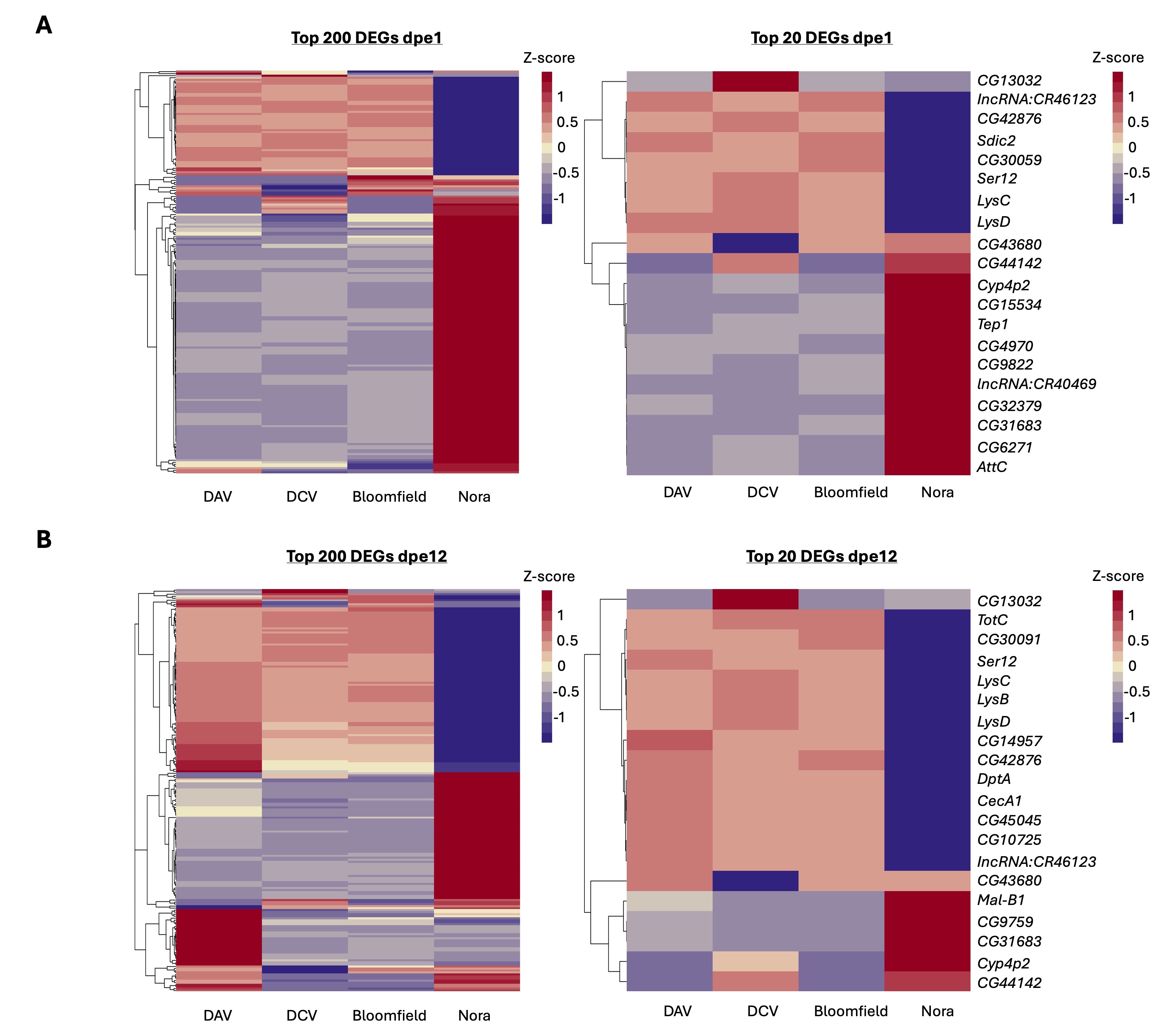

Supplement: S6 Fig — Persistent viral infection alters Drosophila melanogaster’s transcriptomic profile in a virus-independent manner. For all heatmaps, on the X-axis, we indicate the infected stock analysed. A) From left to right: heatmap showing the top 200 differentially expressed genes in our samples in terms of absolute log2 fold change value at dpe1. Heatmap showing the top 20 DEGs at dpe1. B) From left to right: heatmap showing the top 200 differentially expressed genes in our samples in terms of absolute log2 fold change value at dpe12. Heatmap showing the top 20 DEGs at dpe12. For the heatmaps on the right, the top 20 gene names are indicated on the left of the graph (TIF) [file pbio.3003437.s013.tif]

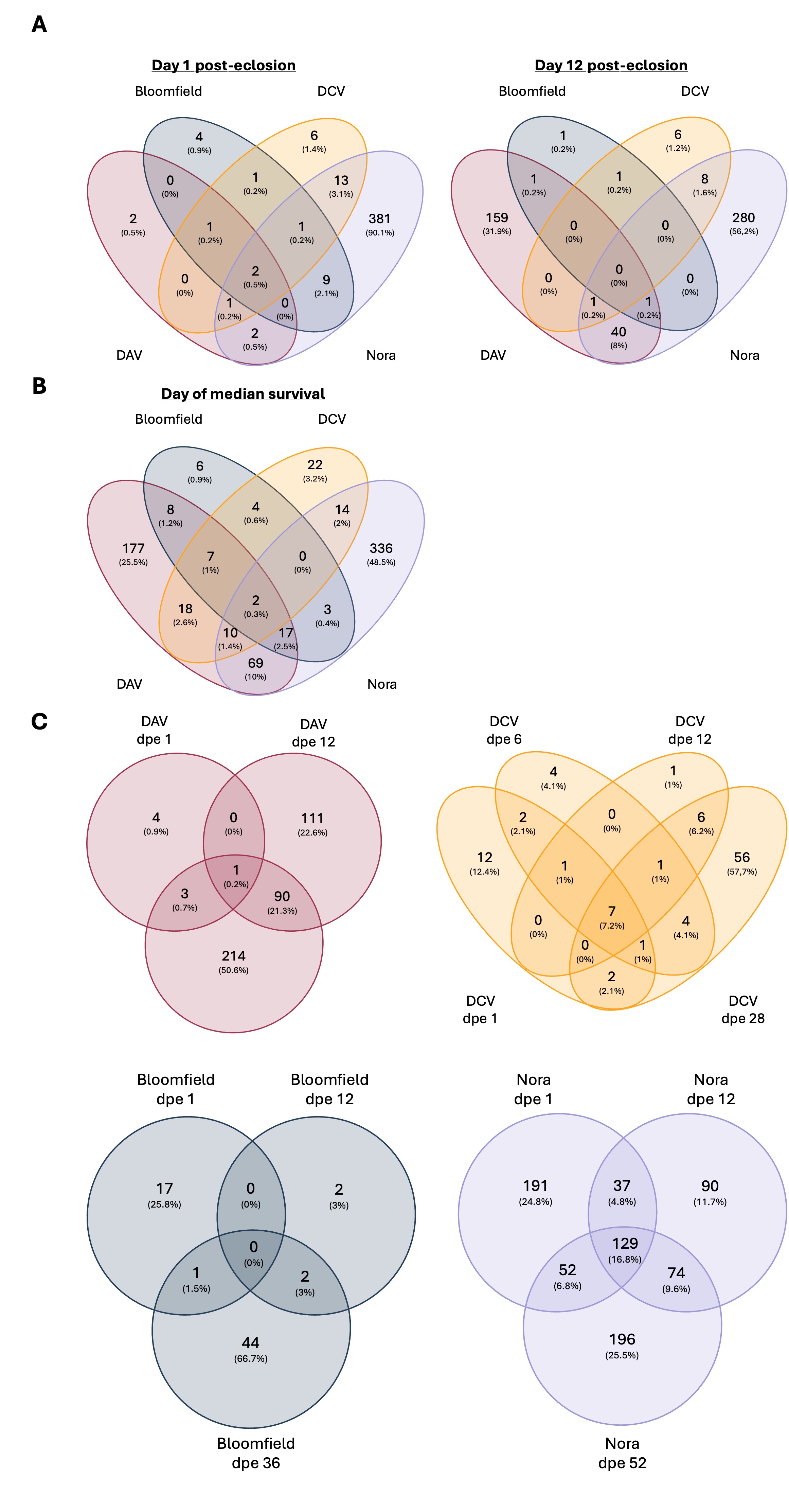

Supplement: S7 Fig — Venn diagrams of differentially expressed genes with differential expression values lower than 1.5 log2 fold change and/or with p-adjusted values lower than 0.05. A) DEGs for days 1, 12, and the day of median survival for each infected individual stock. B) DEGs on the day of median survival of all persistently infected stocks. C) DEGs for each persistently infected stock over time. (TIF) [file pbio.3003437.s014.tif]
