# Supplementary material for: Persistent viral infections impact key biological traits in Drosophila melanogaster
Source: PLoS Biol. 2025 Oct 9;23(10):e3003437. doi: 10.1371/journal.pbio.3003437 (PMC12530575; doi:10.1371/journal.pbio.3003437)
Supplement: Attachment — Submitted filename: REVIEWERS.docx [file pbio.3003437.s017.docx]

Dear Editor and Reviewers,

We thank you for your valuable input. Your concerns have been thoroughly addressed.

Below a point-to-point answer.

**Reviewer #1:**

The paper makes a thorough examination of the consequences of persistent viral infections, using 4 viruses in D. melanogaster, across a range of physiological traits and fitness proxies. The work seems of a high standard and takes a holistic approach, for example measuring viral load and host gene expression and multiple time points during the course of infection. The work is valuable for researchers working on Drosophila virus interactions, and examines infection dynamics and outcomes through this less studied (oral-fecal) route of infection.

The work is a natural progression of the authors previous work, however, it is somewhat incremental. As such the work is lacking in suitable novelty/originality to meet the PLoS Biology publication criteria, and is unlikely to have significance interest outside the field.

We were somewhat surprised by the reviewer’s comment on the overall suitability of the manuscript for this journal. Our understanding is that the purpose of peer review is to evaluate whether the claims are supported by the data, and whether the methods and analyses are appropriate. Assessments of scope and fit are typically left to the editorial team. Such comments may inadvertently diminish the significant contributions of the research community, which we feel is important to acknowledge in order to foster a constructive and supportive scientific environment that treats everyone with respect and fairness.

In response to the concern about “lacking in suitable novelty/originality,” we would like to highlight that, to the best of our knowledge, no previous work has established mono-persistent infections in Drosophila and examined them across development with respect to survival, reproductive output, activity levels, sleep patterns, and transcriptomic responses. In addition, our study provides a valuable transcriptomic resource that will benefit the wider Drosophila virology community.

We are grateful for the reviewer’s remarks and have addressed them carefully, which has led to improvements in the manuscript. At the same time, we would like to emphasize the importance of framing critiques in ways that promote collaboration and uphold the spirit of scientific inquiry. We sincerely thank you for your understanding.

It is not clear why this is a methods and resources paper - whilst the authors have taken an interesting approach I do not feel this meets the PLoS Biology criteria for this type of article; it just seems to be a standard research paper. Methods are at the end and are brief and not at all novel - the major novelty is persistent infections but these are described in a previous paper by the group. In terms of resources, whilst it seems a well-executed and interesting piece of science and the results will be of interest to the field, it does not offer "resources of exceptional and general value" with "applicability and significant utility for the field and spur future research".

Considering the reviewer's remarks, and, in coordination with the editor, the article format is now a Short Article.

Major comments For the study of viable offspring:

- Did you control the density of the populations the parental flies were taken from?

While we did not precisely regulate the number of flies in our parental stocks, we maintain all our stocks at consistent densities. Each fly stock typically consists of 30-40 individuals per tube, and they are transferred to fresh tubes on a weekly basis. 20 males and 20 females from these stocks were used to initiate the offspring experiment.

- Could some of the patterns (eg increased number of offspring in some viral infections) be due to reduced density in infected populations (and so larger flies from this reduced competition)?

As mentioned above, parental stocks are kept at constant density. The number of eggs is calculated from tubes where exactly 20 females and 20 males are kept together for 24 hours, with the 20 females transferred into fresh tubes daily until no more eggs are produced (~17 days). This density has been selected to provide optimal surface area and total tube space to lay eggs, thereby minimizing crowding effects. Of note, few flies die during the total length of the experiment, specially throughout the first days of the assay, and thus death do not significantly influence the density or the outcomes of the assay. The results of the viable offspring assay have now been included in Supplementary File 2. This is now better explained in the manuscript in lines 186-196.

- How was mortality accounted for? Eg should have seen ~25% death in DCV flies by day 10 post infection - were a greater number of offspring produced even if account for female mortality (e.g. could this be life-history trade off with terminal fecundity investment?). This is alluded to in the discussion, but you have the data to formally test, so this should be done. I would encourage some discussion/speculation on transgenerational effects also (e.g. do offspring from infected flies themselves have reduced fitness)

To account for mortality, female mortality was incorporated into the calculations. For each day, the number of alive females was counted in each of the egg-laying tubes. The number of alive females was then established per tube and per day. Each individual tube produced a certain number of viable offspring. The results of the viable offspring assay have now been included in Supplementary File 2. This value was then normalized over the number of females that were alive at the moment of passing the flies onto the new food. Additionally, since most eggs are laid during the initial days, mortality rates are minimal, ensuring that our results remain robust and reliable. This is now better explained in the manuscript, lines 190-196, 671-679.

- For DCV in particular, where you see the greatest mean increase in total offspring (Fig 2b) you also see the greatest variation in this trait. When looking at figure 1 you see a bifurcation in infection outcomes with nearly a quarter of flies being uninfected by day 12, with a bimodal distribution for viral load also. As such I suspect that variation in offspring production may be due to some individuals being infected and some uninfected; although I think this makes the patterns all the more puzzling - this would benefit from some further analysis/thoughts.

Our results show that viral loads can vary significantly among individuals within the same viral stock. This variability likely accounts for the variations observed across experiments using the same stock. Unfortunately, we cannot differentiate between levels of infection amongst live individuals; therefore, our infected fly populations exhibit heterogeneous levels of viral accumulation. This, in turn, can drive the differences observed within the same infected fly stock. This is now better explained in the document (lines 566-574).

RNAseq:

- Supp figure 2 should be in the main manuscript.

The Short Article format has limitations on figure numbers; therefore, this figure will remain in the supplementary section.

- Did you do RNA extractions on individual flies, check their infection status and then pool RNA (ie did you know whether all flies in your pools were infected/uninfected)? This needs clarifying and critical for interpreting DCV infection transcriptome data.

Yes, RNA extractions were performed on individual flies, followed by RT-qPCR to check infection status in each fly. Flies with similar viral RNA accumulation levels were then pooled. We described this better now in lines 345-352.

- Were you mapping reads only to D.mel or to your (and other) viruses as well (eg [https://urldefense.com/v3/__https://doi.org/10.3390/v15091849](https://urldefense.com/v3/__https:/doi.org/10.3390/v15091849) <[https://urldefense.com/v3/__https://doi.org/10.3390/v15091849](https://urldefense.com/v3/__https:/doi.org/10.3390/v15091849)>)?__;!!JFdNOqOXpB6UZW0!tS1XBZmezSOX4AbJ9RwBZ7giHQUz1o9AM-FqF3qYzCpjIbdvLAhJqLQL950Txnr0-ybonc5n2sWtQVqsKF_B$ Helpful for above point and would be interesting to see if any other viruses are hanging around in your populations (an up-to-date list of know Drosophila viruses here [https://urldefense.com/v3/__https://obbard.bio.ed.ac.uk/data.html__;!!JFdNOqOXpB6UZW0!tS1XBZmezSOX4AbJ9RwBZ7giHQUz1o9AM-FqF3qYzCpjIbdvLAhJqLQL950Txnr0-ybonc5n2sWtQThg6LKq$](https://urldefense.com/v3/__https:/obbard.bio.ed.ac.uk/data.html__;!!JFdNOqOXpB6UZW0!tS1XBZmezSOX4AbJ9RwBZ7giHQUz1o9AM-FqF3qYzCpjIbdvLAhJqLQL950Txnr0-ybonc5n2sWtQThg6LKq$) <[https://urldefense.com/v3/__https://obbard.bio.ed.ac.uk/data.html__;!!JFdNOqOXpB6UZW0!tS1XBZmezSOX4AbJ9RwBZ7giHQUz1o9AM-FqF3qYzCpjIbdvLAhJqLQL950Txnr0-ybonc5n2sWtQThg6LKq$](https://urldefense.com/v3/__https:/obbard.bio.ed.ac.uk/data.html__;!!JFdNOqOXpB6UZW0!tS1XBZmezSOX4AbJ9RwBZ7giHQUz1o9AM-FqF3qYzCpjIbdvLAhJqLQL950Txnr0-ybonc5n2sWtQThg6LKq$)> )

The subsection Viral infections (lines 611-638) in Material and Methods has been carefully rewritten to explain how the persistent lines were obtained. In addition, it now states: “In addition, total RNA from persistently infected adults was extracted and sequenced. The resulting reads were mapped to a database of all known Drosophila viruses (https://obbard.bio.ed.ac.uk/data.html) confirming that only the desired virus was present (Nigg et al., 2022, 2024).”

L476 - plot and test the trade-off (should be a negative correlation) between survival and reproductive output

The reviewer is correct. Figure 1E is presents the negative correlation between survival and reproductive otuput.

Minor comments

Abstract - remove "challenge the assumption of the innocuousness of persistent viral infections for the host" - it is not a widely held assumption that persistent infections are benign.
We have removed this and another misleading mentions.

First paragraph of the intro seems rather narrow in breadth.

- Firstly I would suggest incorporating some of the large scale sequencing studies for virus discovery eg [https://urldefense.com/v3/__https://doi.org/10.1016/j.cell.2024.09.027__;!!JFdNOqOXpB6UZW0!tS1XBZmezSOX4AbJ9RwBZ7giHQUz1o9AM-FqF3qYzCpjIbdvLAhJqLQL950Txnr0-ybonc5n2sWtQZBTqhQB$](https://urldefense.com/v3/__https:/doi.org/10.1016/j.cell.2024.09.027__;!!JFdNOqOXpB6UZW0!tS1XBZmezSOX4AbJ9RwBZ7giHQUz1o9AM-FqF3qYzCpjIbdvLAhJqLQL950Txnr0-ybonc5n2sWtQZBTqhQB$) <[https://urldefense.com/v3/__https://doi.org/10.1016/j.cell.2024.09.027__;!!JFdNOqOXpB6UZW0!tS1XBZmezSOX4AbJ9RwBZ7giHQUz1o9AM-FqF3qYzCpjIbdvLAhJqLQL950Txnr0-ybonc5n2sWtQZBTqhQB$](https://urldefense.com/v3/__https:/doi.org/10.1016/j.cell.2024.09.027__;!!JFdNOqOXpB6UZW0!tS1XBZmezSOX4AbJ9RwBZ7giHQUz1o9AM-FqF3qYzCpjIbdvLAhJqLQL950Txnr0-ybonc5n2sWtQZBTqhQB$)> and [https://urldefense.com/v3/__https://www.nature.com/articles/nature20167__;!!JFdNOqOXpB6UZW0!tS1XBZmezSOX4AbJ9RwBZ7giHQUz1o9AM-FqF3qYzCpjIbdvLAhJqLQL950Txnr0-ybonc5n2sWtQUDrXHnc$](https://urldefense.com/v3/__https:/www.nature.com/articles/nature20167__;!!JFdNOqOXpB6UZW0!tS1XBZmezSOX4AbJ9RwBZ7giHQUz1o9AM-FqF3qYzCpjIbdvLAhJqLQL950Txnr0-ybonc5n2sWtQUDrXHnc$) <[https://urldefense.com/v3/__https://www.nature.com/articles/nature20167__;!!JFdNOqOXpB6UZW0!tS1XBZmezSOX4AbJ9RwBZ7giHQUz1o9AM-FqF3qYzCpjIbdvLAhJqLQL950Txnr0-ybonc5n2sWtQUDrXHnc$](https://urldefense.com/v3/__https:/www.nature.com/articles/nature20167__;!!JFdNOqOXpB6UZW0!tS1XBZmezSOX4AbJ9RwBZ7giHQUz1o9AM-FqF3qYzCpjIbdvLAhJqLQL950Txnr0-ybonc5n2sWtQUDrXHnc$)>
- Secondly this would benefit from citing some studies other than the authors own on persistent viral infections to show a wide view on this type of infection

We agree with the reviewer’s comments and have expanded this section with the following references:

- Hou, X., He, Y., Fang, P., Mei, S.-Q., Xu, Z., Wu, W.-C., Tian, J.-H., Zhang, S., Zeng, Z.-Y., Gou, Q.-Y., Xin, G.-Y., Le, S.-J., Xia, Y.-Y., Zhou, Y.-L., Hui, F.-M., Pan, Y.-F., Eden, J.-S., Yang, Z.-H., Han, C., … Shi, M. (2024). Using artificial intelligence to document the hidden RNA virosphere. Cell, 187(24), 6929-6942.e16. https://doi.org/10.1016/j.cell.2024.09.027
- Lee, W.-S., Webster, J. A., Madzokere, E. T., Stephenson, E. B., & Herrero, L. J. (2019). Mosquito antiviral defense mechanisms: A delicate balance between innate immunity and persistent viral infection. Parasites & Vectors, 12(1), 165. https://doi.org/10.1186/s13071-019-3433-8
- Obbard, D. J., Shi, M., Roberts, K. E., Longdon, B., & Dennis, A. B. (2020). A new lineage of segmented RNA viruses infecting animals. Virus Evolution, 6(1), vez061. https://doi.org/10.1093/ve/vez061
- Shi, M., Lin, X.-D., Tian, J.-H., Chen, L.-J., Chen, X., Li, C.-X., Qin, X.-C., Li, J., Cao, J.-P., Eden, J.-S., Buchmann, J., Wang, W., Xu, J., Holmes, E. C., & Zhang, Y.-Z. (2016). Redefining the invertebrate RNA virosphere. Nature, 540(7634), 539-543. https://doi.org/10.1038/nature20167
- Webster, C. L., Longdon, B., Lewis, S. H., & Obbard, D. J. (2016). Twenty-Five New Viruses Associated with the Drosophilidae (Diptera). Evolutionary Bioinformatics, 12s2, EBO.S39454. <https://doi.org/10.4137/EBO.S39454>
- Webster, C. L., Waldron, F. M., Robertson, S., Crowson, D., Ferrari, G., Quintana, J. F., Brouqui, J.-M., Bayne, E. H., Longdon, B., Buck, A. H., Lazzaro, B. P., Akorli, J., Haddrill, P. R., & Obbard, D. J. (2015). The Discovery, Distribution, and Evolution of Viruses Associated with Drosophila melanogaster. PLOS Biology, 13(7), e1002210. https://doi.org/10.1371/journal.pbio.1002210

L479 - delete "to ensure population survival" - this reads as some slightly odd group selectionist argument which isn't needed.

Done.

L499 - 500 - cite a few more papers here to demonstrate the large amount of info on genetic variation in susceptibility to virus infection

We have extended with the following references:

- Cogni, R., Cao, C., Day, J. P., Bridson, C., & Jiggins, F. M. (2016). The genetic architecture of resistance to virus infection in Drosophila. Molecular Ecology, 25(20), 5228-5241. https://doi.org/10.1111/mec.13769
- Kutzer, M. A. M., Gupta, V., Neophytou, K., Doublet, V., Monteith, K. M., & Vale, P. F. (2023). Intraspecific genetic variation in host vigour, viral load and disease tolerance during Drosophila C virus infection. Open Biology, 13(3), 230025. https://doi.org/10.1098/rsob.230025

More generally I felt there could be better scholarship and referencing of the literature, seemed to be picking just a couple of relevant examples rather than citing a broader range of relevant studies.

We have reviewed the use of literature in the text, and expanded the sources used.

L510-511 add note saying lab microbiome probably not typical of wild flies

We agree that the distinction should be done. This has now been added to the discussion (lines 504-505).

L588 - delete "comparative data"

Done.

Legends on supplementary figures need expanding as atm it is hard to tell (without ref back to main ms) which data they are referring too.

These changes have been adapted in the figure captions.

Data availability - an SRA number was provided but this data was not released. Data and code for non-sequencing data and analysis was also not available.

The data is now publicly available.

**Reviewer #2:**

In this manuscript, the authors investigated the impact of four persistent viral infections on several traits in Drosophila. All four viruses reduced the survival of flies but reached different load and prevalence in fly populations: DAV was present in all individuals, while DCV was cleared by most of the flies. The authors detected the effect of viruses on offspring production, sleep duration, and locomotor activity. Microbiome composition was not significantly affected by persistent viral infections, however total bacterial load significantly altered. Finally, the authors performed transcriptomic analysis of infected flies and different time-points and found that it is virus-specific and can persist even after clearence of the virus. Given a limited information on the impact of persistent infections on the host, this study will be of great general interest but also a valuable resource for Drosophila researchers. Overall, this is a well-performed and clearly-written work.

I have few suggestions to enhance the usability of data generated in this work.

1. Since this is a Resources paper, the authors should make their data as accessible and usable as possible. This is not the case for RNA-seq results. If someone wants to check if and how any gene changes expression under studied conditions, this is not possible to do with the information provided. The authors should provide all the transcriptomics results (tables with genes, their expression, and statistics) as supplementary files.

We confirm that the RNA-seq data is now available.

2. Volcano plots (sup fig 3). It would be useful to show at least for the most significant dots which genes stand behind them (like in fig 7b).

We have now added the names to the top 10 differentially expressed genes (in terms of absolute Log_2_FC value).

3. Can the authors please provide justification as to why they used whole fly samples and not guts for their RNAseq, given that infections are intestinal?

Persistent infections with enteric viruses are transmitted and sustained primarily via the oral-fecal route. The gut serves as the primary infection site, however with time, the virus spread systemically (see Nigg et al, 2024, DAV level in carcasses). Therefore, we used the whole fly sample as we follow infections on time. We agree with the reviewer that for a future work, it would be interesting to concentrate in early times post infection and produce transcriptomic data only from the gut.

- Nigg JC, Castelló-Sanjuán M, Blanc H, Frangeul L, Mongelli V, Godron X, et al. Viral infection disrupts intestinal homeostasis via Sting-dependent NF-κB signaling in Drosophila. Curr Biol. 2024; 34: 2785-2800.e7. doi:10.1016/j.cub.2024.05.009

4. L604. Diet composition should be provided.

Description of the diet composition is now provided in materials and methods (lines 604-609).

5. L607-610. It should be described how infections were established.

The subsection Viral infections (lines 611-638) in Material and Methods has been carefully rewritten to explain how the persistent lines were obtained.

**Reviewer #3:**

In their paper, "Persistent viral infections impact key biological traits in Drosophila melanogaster", Castello-Sanjuan et al. show that persistent infection by four different viruses results in significant, virus-specific changes in viral titer, longevity, lifetime fecundity, locomotion, sleep time, microbial load in the digestive tract, and the whole body transcriptome. The experiments are generally well done, and the manuscript is well-written. Given its comprehensiveness and breadth, the study provides a novel and important resource to the field of host-microbe interactions and infectious disease. I commend the authors on the substantial amount of work that went into this study and the contribution they are making to the field. I have some concerns that should be addressed before publication, however, and they are outlined in detail below.

Major concerns:

1. Essential: The authors do not indicate whether the method used to establish persistent viral infection is representative of how flies would become persistently infected in a natural habitat. Moreover, the authors do not state whether persistent infection is common in natural populations for any of these viruses. Therefore, it is unclear whether this method to induce persistent infection is relevant to the natural ecology or evolution of any of these virus-host systems. This would not be an issue if the authors were solely trying to investigate the physiological effects of persistent infection on flies, but they attempt to connect these effects to the evolution of these viruses in natural populations. For these arguments to stand, they must make it clear whether their experimental setup in any way recapitulates naturally acquired persistent infection. Related to this question, assuming persistent infection is common in natural populations (which needs to be established and clearly explained by the authors), are the viral titers found in flies using this method similar to what would be found in a naturally infected fly with persistent infection?

Our study describes persistent viral infections that mimic natural transmission patterns. These enteric viruses, first identified in wild Drosophila populations and lab fly stocks, naturally establish infections that are maintained mainly through horizontal oral-fecal transmission. Unlike injection methods that artificially bypass intestinal barriers, our approach ensures a more natural-like exposure: flies are infected orally by feeding on infected food, simulating environmental acquisition. The gut is the site of initial infection, although infections eventually become systemic.

We have now given more details on the method we used to establish persistent infections (lines 611-638).

Regarding viral loads, natural populations exhibit substantial heterogeneity in infection levels due to multiple factors, including host genetics, environmental conditions, and co-infection status (Webster et al., 2015). While no laboratory system can perfectly replicate the full complexity of natural ecosystems, our model captures the essential features of persistent infection establishment and maintenance observed in wild populations. The viral titers we observe fall within ranges documented in naturally infected flies, though we acknowledge that laboratory conditions may influence absolute load levels.

- Webster, C. L., Waldron, F. M., Robertson, S., Crowson, D., Ferrari, G., Quintana, J. F., Brouqui, J.-M., Bayne, E. H., Longdon, B., Buck, A. H., Lazzaro, B. P., Akorli, J., Haddrill, P. R., & Obbard, D. J. (2015). The Discovery, Distribution, and Evolution of Viruses Associated with Drosophila melanogaster. PLOS Biology, 13(7), e1002210. https://doi.org/10.1371/journal.pbio.1002210

2. How does evolution of the viral population potentially compromise the findings? Since the virus is being perpetually propagated among individuals from the same genetic background, is it possible it has adapted in a manner that is no longer representative of how wild viruses would impact the fitness of wild flies? What differences might be expected if the authors were to use recently isolated viruses? Again, the primary reason this is of concern is the stated implications of the findings for the evolution of these virus-host systems.

We acknowledge potential limitations regarding laboratory viral evolution. However, recently isolated viral strains could also be very well adapted to their hosts, as these viruses naturally persist in wild Drosophila populations where they have undergone extensive coevolution. The distinction between "laboratory-adapted" and "wild" viruses may be less meaningful than assumed, since natural viral populations are already highly adapted to their Drosophila hosts through ongoing evolutionary processes in the wild.

Even if laboratory propagation has altered some viral characteristics, our core findings about fitness impacts would remain relevant. The fundamental biological constraints we document (that persistent infections impose significant costs on survival and reproduction) reflect general principles of host-pathogen interactions rather than artifacts of specific viral strains.

3. Essential: When measuring total offspring per female, the females were transferred to new food every day. Therefore, it seems likely to this reviewer that the persistence of viral infection would differ from that of the standard protocol used in this study (which was to transfer flies to new vials every two days). Can the authors speak to whether they have any data on persistence of viral infection in females during the fecundity experiment? If not, this should be presented as a potential caveat to the study.

The reviewer is right, we have not considered this possibility and therefore, we included this caveat into the discussion. See lines 480-485 and lines 574-577.

To answer to the question of the reviewer, we don’t have currently any data on persistence of viral infection in females that are transferred daily. However, because the infection prevalence in the parental stock is almost total, we don’t see how daily transferring the females to new tubes could clean the infection. Furthermore, we have described in previous work (Nigg et al, 2022) that to establish persistently infected lines, flies have to be in contact with the virus since the larvae stage.

4. Essential: The authors refer to Lactobacillus throughout the paper, but the most common OTU in their data set was assigned to Lactiplantibacillus (formerly Lactobacillus) which as far as I can tell, has been determined to be a separate genus from its former designation of Lactobacillus. See here for associated publications: [https://urldefense.com/v3/__https://lpsn.dsmz.de/genus/lactiplantibacillus__;!!JFdNOqOXpB6UZW0!tS1XBZmezSOX4AbJ9RwBZ7giHQUz1o9AM-FqF3qYzCpjIbdvLAhJqLQL950Txnr0-ybonc5n2sWtQaYZGiNK$](https://urldefense.com/v3/__https:/lpsn.dsmz.de/genus/lactiplantibacillus__;!!JFdNOqOXpB6UZW0!tS1XBZmezSOX4AbJ9RwBZ7giHQUz1o9AM-FqF3qYzCpjIbdvLAhJqLQL950Txnr0-ybonc5n2sWtQaYZGiNK$) <[https://urldefense.com/v3/__https://lpsn.dsmz.de/genus/lactiplantibacillus__;!!JFdNOqOXpB6UZW0!tS1XBZmezSOX4AbJ9RwBZ7giHQUz1o9AM-FqF3qYzCpjIbdvLAhJqLQL950Txnr0-ybonc5n2sWtQaYZGiNK$](https://urldefense.com/v3/__https:/lpsn.dsmz.de/genus/lactiplantibacillus__;!!JFdNOqOXpB6UZW0!tS1XBZmezSOX4AbJ9RwBZ7giHQUz1o9AM-FqF3qYzCpjIbdvLAhJqLQL950Txnr0-ybonc5n2sWtQaYZGiNK$)> Unless I am misunderstanding something, the authors should make this correction.

Thanks for spotting this. We corrected bacterial taxonomy accordingly (lines 254 and 750).

5. Essential: It is not clear to this reviewer why PCR amplification followed by sequencing failed to yield any sequences in Bloomfield infected flies, but qPCR was successful. Given that both approaches have an amplification step, why was it successful for one but not the other? The authors don't mention negative controls in the methods for their microbiota sequencing or qPCR. I assume they were used but this should be clearly stated. For qPCR, please clarify how the negative controls were used to distinguish true signal from contamination. For example, indicating that the negative controls yielded no signal or that the signal obtained from the negative controls was sufficiently weaker than that in Bloomfield flies. Otherwise, because of the ubiquity of contaminating 16S DNA in any laboratory, it is impossible to know if the Bloomfield signal is real or just environmental contamination.

We thank the reviewer for this helpful comment, which allowed us to clarify the results of this section. We consider that the different amplification outcomes between the full-length 16S PCR (used for microbiota composition analysis) and the 16S qPCR in Bloomfield virus-infected flies are attributable to the different amplicon sizes (1.5 Kb vs ~ 200 bp). Specifically, we were unable to amplify the full 16S fragment from DNA extracted from Bloomfield virus-infected flies, and no amplification was observed in extraction negative controls. In contrast, 16S qPCR produced Ct values in the Bloomfield samples were lower than no-template controls, but comparable to extraction negative controls, suggesting that these signals likely reflect low-level laboratory contaminants rather than the actual fly microbiota. We have therefore revised the text to state that Bloomfield virus-infected flies harbor microbiota loads below the limit of detection and change the figure accordingly (Figure 1G, **results**: lines 255-265, **methods**: lines 717-718, 724, 761-762).

6. Essential: In general, pooling individuals for microbiota sequencing in species with environmentally acquired microbiota is not ideal, because there can be substantial inter-individual variation and a single individual with a high amount of one OTU can skew the entire pool, resulting in incorrect conclusions. I know pooling is common practice in Drosophila, perhaps because of limited inter-individual variation in microbiota composition, but I suggest moving away from this practice whenever time and funding allows. I note that pools are not used by the authors in any other assay (except for transcriptomics), therefore I assume they recognize the importance of accounting for inter-individual variation. I do not suggest the authors need to collect more data for this study, but including a comment and supporting citation(s) in the methods justifying the approach of pooling or discussing its limitations is called for.

We somehow disagree with this comment. We believe that inter-individual variation is important in most biological questions, however insects that are reared together are very unlikely to show relevant inter-individual variation in microbiota composition, due to the shared genotype, environment, and diet. Drosophila is characterized by a low diverse microbiota to a point that most lab colonies carry exclusively *Lactiplantibacillus-Levilactobacillus* and *Acetobacter* bacteria (see Battistolli et al., 2025; Broderick & Lemaitre, 2012; Marra et al., 2021; Wong et al., 2011). The fact that our persistently infected fly lines (thus flies reared independently) have similar microbiota composition further corroborate this low variability. All Drosophila studies investigating the microbiota composition of lab flies use pools of flies (see Broderick & Lemaitre, 2012, in which most studies used from 7 to 100 flies per pool), possibly to overcome the technical limitation of amplifying such low microbial loads. We added a sentence in the method section to justify the use of pooled flies (lines 713-715).

- Battistolli, M., Varponi, I., Romoli, O., & Sandrelli, F. (2025). The circadian clock gene period regulates the composition and daily bacterial load of the gut microbiome in Drosophila melanogaster. Scientific Reports, 15(1), 1016. https://doi.org/10.1038/s41598-024-84455-4
- Broderick, N. A., & Lemaitre, B. (2012). Gut-associated microbes of Drosophila melanogaster. Gut Microbes, 3(4), 307-321. https://doi.org/10.4161/gmic.19896
- Marra, A., Hanson, M. A., Kondo, S., Erkosar, B., & Lemaitre, B. (2021). Drosophila Antimicrobial Peptides and Lysozymes Regulate Gut Microbiota Composition and Abundance. mBio, 12(4), e00824-21. https://doi.org/10.1128/mBio.00824-21
- Wong, C. N. A., Ng, P., & Douglas, A. E. (2011). Low‐diversity bacterial community in the gut of the fruitfly Drosophila melanogaster. Environmental Microbiology, 13(7), 1889-1900. https://doi.org/10.1111/j.1462-2920.2011.02511.x

7. Essential: At what taxonomic level were the microbiota diversity analyses performed? This should be indicated in the methods. Also, what variables were included in the PERMANOVA? To what variable does the p-value provided refer?

Microbiota diversity analyses were performed at the OTU level. PERMANOVA was performed on the Bray-Curtis dissimilarity matrix and included exclusively the infection status as variable, thus the p value refers to the “Infection” variable. We included this information in the text (legend of Supplementary Figure 3 and lines 252-2453).

Minor concerns:

Line 717 OTU, not OUT.

Done.

The authors refer to OTU classification in the methods but bacterial species throughout the study. Was species level ID obtained for the vast majority of OTUs? If not, OTU would probably be a more appropriate term to use.

We changed “species” with “OTU” (legend of Supplementary Figure 3 and lines 746, 750).

Figure 5, the legend indicates that "Shape fill indicates the replicate" but there are only two shape fills and each is used for three data points. Therefore, I am confused as to what shape fill is indicating and the general replication structure of the qPCR data.

To clarify this, the figure legend (now part of Figure 1) was modified as follows: “The experiment was performed twice, with three pools per line analyzed in each experiment… Shape fill indicates the replicate”.

Line 529: The authors refer to "The systemic impact…" but don't explain what they are referring to here? Is the assumption that the impact of infection on locomotion is a systemic impact because it's outside the gut? Please make the connection more clear, as it was confusing the first time I read through it.

Flies are infected by oral acquisition of viruses, mimicking natural environmental acquisition. The gut is the site of initial infection, although the infection eventually becomes systemic. This has been previously reported by Nigg et al (2024; DAV infection levels differ between the gut vs the carcass). The dissemination of the infection from the gut to other tissues has effects beyond the gut in host fitness.

- Nigg JC, Castelló-Sanjuán M, Blanc H, Frangeul L, Mongelli V, Godron X, et al. Viral infection disrupts intestinal homeostasis via Sting-dependent NF-κB signaling in Drosophila. Curr Biol. 2024; 34: 2785-2800.e7. doi:10.1016/j.cub.2024.05.009

Supplementary Figure 1: Why does Uninfected have error bars but no other treatment? Please make this uniform.

Uninfected flies have error bars because their Chao1 index has standard error, while for all other lines the standard error is equal to zero. Below the table of bacteriome diversity analysis:

|  | Infection | Replicate | Ordine | samples | variable | value | se |
| --- | --- | --- | --- | --- | --- | --- | --- |
| 1 | DAV | 1 | D | DAV_1 | Chao1 | 21 | 0 |
| 2 | DAV | 2 | E | DAV_2 | Chao1 | 21 | 0 |
| 3 | DAV | 3 | F | DAV_3 | Chao1 | 17 | 0 |
| 4 | DCV | 1 | G | DCV_1 | Chao1 | 7 | 0 |
| 5 | DCV | 2 | H | DCV_2 | Chao1 | 11 | 0 |
| 6 | DCV | 3 | I | DCV_3 | Chao1 | 16 | 0 |
| 7 | Nora | 1 | J | Nora_1 | Chao1 | 7 | 0 |
| 8 | Nora | 2 | K | Nora_2 | Chao1 | 20 | 0 |
| 9 | Nora | 3 | L | Nora_3 | Chao1 | 17 | 0 |
| 10 | Uninfected | 1 | A | Uninfected_1 | Chao1 | 26 | 13.129532 |
| 11 | Uninfected | 2 | B | Uninfected_2 | Chao1 | 19.2 | 4.327688 |
| 12 | Uninfected | 3 | C | Uninfected_3 | Chao1 | 20.6 | 5.33279 |
| 13 | DAV | 1 | D | DAV_1 | Shannon | 0.69930539 | NA |
| 14 | DAV | 2 | E | DAV_2 | Shannon | 0.52319347 | NA |
| 15 | DAV | 3 | F | DAV_3 | Shannon | 0.31736 | NA |
| 16 | DCV | 1 | G | DCV_1 | Shannon | 0.50723717 | NA |
| 17 | DCV | 2 | H | DCV_2 | Shannon | 0.09591743 | NA |
| 18 | DCV | 3 | I | DCV_3 | Shannon | 0.66049964 | NA |
| 19 | Nora | 1 | J | Nora_1 | Shannon | 0.76637963 | NA |
| 20 | Nora | 2 | K | Nora_2 | Shannon | 0.43108805 | NA |
| 21 | Nora | 3 | L | Nora_3 | Shannon | 0.47656216 | NA |
| 22 | Uninfected | 1 | A | Uninfected_1 | Shannon | 0.7023284 | NA |
| 23 | Uninfected | 2 | B | Uninfected_2 | Shannon | 0.70458352 | NA |
| 24 | Uninfected | 3 | C | Uninfected_3 | Shannon | 0.70393631 | NA |

**Reviewer #4:**

This manuscript presents a detailed study of the effects of four viruses causing persistent infections in the fitness, behavior, transcriptomic patterns and microbiome of their host Drosophila melanogaster. Although persistent infections are not uncommon, most studies focused on acute viruses. Thus, this study is scientifically relevant and timely as it tackles an important biological question, yet only partially understood. The manuscript is well written and easy to follow. The data presentation helps the reader navigate through the breath of experiments done and support the conclusions of the work. Thus, I think this is a neat piece of science and I only have some minor comments:

- Figure 1c. I’m not sure of understanding the pie diagrams. According to panel 1b, all viruses induce certain level of mortality from 20 dpe on, or even earlier. If so, the number of flies analyzed in panel 1c should not be constant over time as certain mortality is expected and prevalence should be calculated over surviving individuals. Were all flies surviving until 28 (or even 52) dpe? If so, how does this result fit with those on panel 1b?

The survival curve (Figure 1B) illustrates the survival curve of the different infected stocks over time and indicates the median survival days. Using this information, flies were sampled on the days shown in Figure 1A. These adult female fly samples were alive at the time of collection. Therefore, mortality should not be considered in the viral load dynamics section (Figure 1E). In the pie charts (bottom panel of figure 1E) we show the prevalence of viral RNA in the collected samples (i.e. number of infected individuals over the total). The numbers below the pie charts indicate how many of the total collected flies are infected.

- The authors quantify effects on host fitness as production of viable progeny per female. This is clearly a good proxy and I am not criticizing it. However, in various parts of the manuscript the authors mention that virus-induced changes in host fitness have consequences for the host population. To address this question, I think that, rather that descendants per female, the relevant parameter is the total progeny production per population (per tube or per treatment in this case). This measure should take into account not only the total number of descendants produced per female, but also how many females survived to produce progeny and the prevalence of the virus in the population (i.e., the total number of descendants in the population will be the sum of that produced by infected and non-infected individuals. Did the authors quantify this parameter? It should be quite straightforward as all the necessary information is already available.

We fully agree that population-level fitness consequences require accounting for both reproductive output and adult survival prevalence. For these purposes we have now included the total population offspring production (now Figure 1C). This graph presents the total number of adult flies emerging from daily offspring production, normalized to account for fly mortality. Unlike a simple depiction of egg-laying patterns over time post-mating, it illustrates the cumulative count of viable offspring emerging each day per female. This normalized cumulative measure enables a more accurate assessment of offspring production dynamics, effectively incorporating the impact of fly mortality and providing a comprehensive view of reproductive output. Moreover, to further include the effects of viral infection on fly survival and offspring production, we have added the new Figure 1D. In this figure, we have plotted the total offspring per female over the median survival day of each infected stock. This new analysis confirms that there is a negative correlation between offspring production and survival.

- Lines 476-485. The authors invoke a survival-fitness trade-off to explain their results. However, some of the results might not be in line with this conclusion. First, all hosts (infected or not) produced progeny in the first 12-15 dpe. Except for DCV, virus-induced mortality is negligible at this point. Thus, virus infection is apparently affecting survival after progeny is produced. Second, the authors discus that higher progeny production comes at the cost of lower immunity, which would explain higher mortality of infected individuals. If so, I would expect those viruses inducing higher progeny production to multiply to higher levels in the host as immunity would be weaker. Nevertheless, DCV and Bloomfield, which induce the highest increases in progeny production are also those with lower virus multiplication levels, suggesting an active immune response. An alternative explanation for these results is that the virus adapted to remain at low levels in order to allow progeny production such that its chances for transmission are enhanced (i.e., the population of susceptible hosts is larger). I would like to know the authors opinion about this possibility.

See our answer above and the new Figure 1D regarding this concern.

- Lines 488-490. If viruses induce accelerated aging, would not shorter reproductive periods be expected?

This important factor was considered when designing the experiment. By limiting mating events to the first 24 hours of adult flies, we reduce the effects of advanced infection, such as accelerated aging, in our progeny assays. Since all flies are at the same stage and the adults have not had time to live longer, all effects originate from their previous infection status during pupation and early adulthood. This experimental design does not account for sustained reproduction over time.

- A curiosity. I came across recently over several papers showing that genes traditionally considered as good normalizers of virus titer are indeed affected by the virus presence. Did the authors check that Rp49 expression was not affected by virus infection?

Yes, Ct values of the house keeping genes were similar across all infected populations as well as uninfected control.

- Finally, I detected some typos: (i) Line 41, species name should be in italics. (ii) Fig 2a, please review the x axis, values 14 and 15 overlap. (iii) Line 522, there is a dot before the reference.

The typos have been fixed.
